# Individualized surgical treatment using decellularized fish skin transplantation after enzymatic debridement: A two years retrospective analysis

**DOI:** 10.1016/j.jpra.2024.07.013

**Published:** 2024-10-18

**Authors:** Gerrit Freund, Benedikt Schäfer, Justus P. Beier, Anja M. Boos

**Affiliations:** Department of Plastic Surgery, Hand Surgery–Burn Center, University Hospital RWTH Aachen, Pauwelsstraße 30, 52074 Aachen, Germany

**Keywords:** Enzymatic Debridement, Decellularized Fish Skin, Treatment of mixed depth burns

## Abstract

Over the past few years, treatment of burn injuries has evolved beyond primary surgical therapy with the development of enzymatic debridement and new types of skin replacement materials by providing complex personalized therapy concepts aimed at preserving and replacing the dermal layer of the skin.

The aim of our study was to develop an individualized treatment algorithm for mixed depth burn wound and evaluate the outcomes of individualized combined treatment of mixed depth burn wounds with enzymatic debridement and decellularized fish skin.

A total of 18 patients with a mean age of 34.8 years and mean follow-up of 447.6 days were included. The mean total burn surface area was 12.3%. All patients received enzymatic debridement and an average area of 247.2 cm^2^ of decellularized fish skin. Days until complete epithelization were 49.4 ± 25.79 days. No patient developed scar contracture or keloid. The Patient and Observer Scar Assessment Scale (POSAS) observer scale showed an overall impression average of 2.2 ± 0.83. The POSAS patient scale showed an overall impression average 2 ± 0.7. The Vancouver Scar Scale showed an average score of 1.89 ± 1.45. In conclusion, combined treatment using enzymatic debridement and decellularized fish skin, polylactide membrane, or split skin grafts allows for a more individualized therapy for mixed depth burn wounds. Fish skin was found to provide a satisfying result in terms of the overall outcome of the developed scar tissue and could lead to a reduction in the area that requires autologous transplantation.

## Introduction

Thermal injuries are among the most serious injuries and their consequences are one of the most lasting traumatic experiences in children and adults. This results in high demand on the quality of care, which requires selective and individualized treatment. Impaired wound healing due to systemic inflammatory reaction presents one of the key problems in surgical treatment of burns. This inflammatory reaction is an early debridement and a temporary wound coverage.^1^ Standard of care for deep burn wounds is nonselective eschar removal using a Humby or Weck skin knife followed by split skin grafting. Over the past few years, the treatment of burn injuries has developed beyond primary surgical therapy with the development of enzymatic debridement and new types of skin replacement materials providing complex personalized therapy concepts.^2^ Large area burns with limited donor areas and mixed burns with the inherent risk of removing vital tissue are particularly challenging. Selective enzymatic debridement considerably reduces the time from the injury to complete debridement compared to conventional surgical excision. Furthermore, blood loss is significantly decreased during eschar removal compared to surgical excision.^3^ The use of a selective enzymatic debridement also decreases the need for autologous skin grafting and the number of wounds requiring further surgical excision by preserving more viable dermis.^4^ Compared to surgical excision, enzymatic debridement in burn treatment reduces the rate of wound infections and can decrease the duration of hospitalization.^5^

In addition, enzymatic debridement can reduce surgical procedure time and operation room capacity.^6^

Decellularized fish skin from Atlantic cod has already demonstrated its potential in the healing of chronic and burn wounds in clinical use.^7^ Compared to other xenogeneic matrices, there are no known infectious diseases that can be transmitted from Atlantic cod to humans.^8^ In addition, the biologically high proportion of omega 3 fatty acids has antibacterial, antiviral, and anti-inflammatory properties and electron microscopic examinations have revealed a similarity in its dermal structure to that of the human dermis, which supports cell migration, proliferation, and in-growth. Thus, decellularized fish skin appears to be potentially promising for the treatment of deep dermal burn wounds.^9^^,^^10^ Differences in pigmentation in the exposed areas, instability of healed skin, secondary healing, and scarring after treatment remain major long-term problem for patients with severe burn injuries. Furthermore, these injuries cause contractions and restrict movement.^11^^,^^12^

The aim of this study was to develop and individualized a treatment algorithm for mixed depth burn wounds and evaluate the outcomes of an individualized combined treatment of mixed depth burn wounds using enzymatic debridement (NexoBrid™, MediWound Germany GmbH, Rüsselsheim, Germany) and decellularized fish skin (Kerecis® Omega3 Wound, Isafjordur, Iceland).

## Material and Methods

### Selection of Subjects

Data from patients with mixed dermal burn wounds receiving enzymatic debridement followed by wound coverage with decellularized fish skin were collected retrospectively. Written consent was obtained from all patients.

**Fig. 1:**
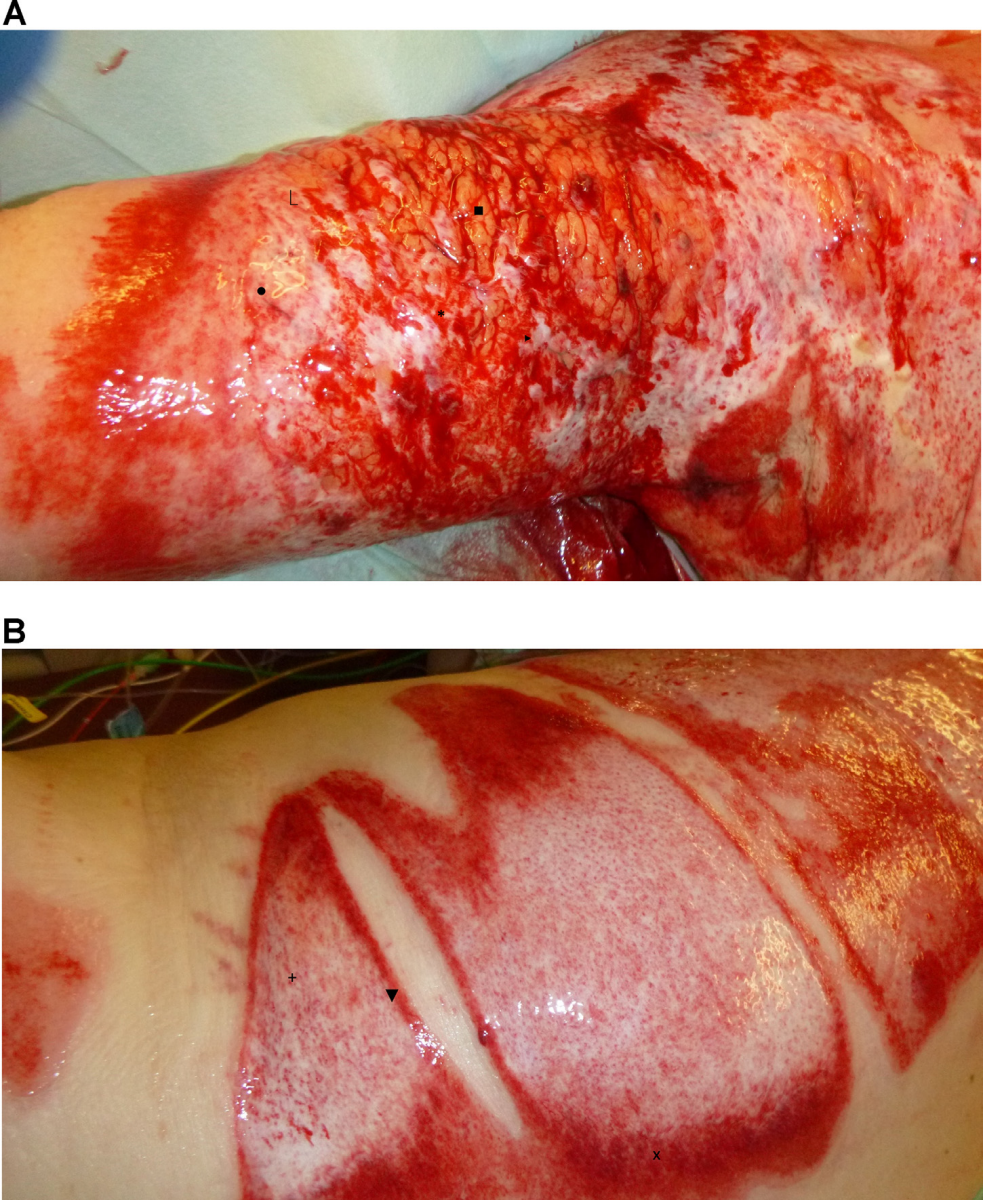
Wound assessment after 4 h of enzymatic debridement (A and B). The depth of the wound depends on the bleeding pattern. ● deep dermis ⎿ large visible step * very deep dermis ■ subcutaneous fat ▸visible vessels + deep reticular dermis □ reticular dermis x papillar ▼ small visible step

**Fig. 2:**
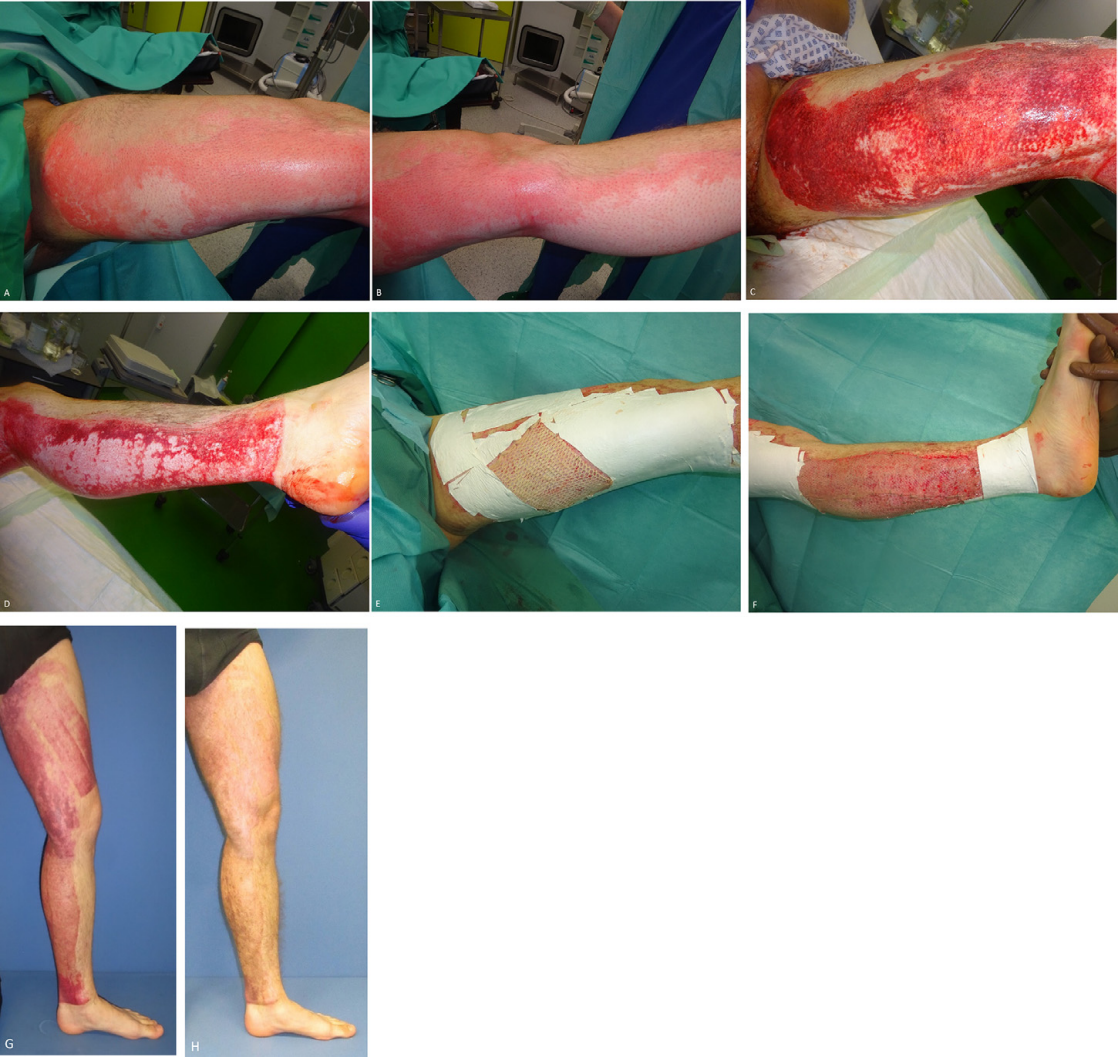
A, B) Intraoperative findings after blister removal; C, D) findings after enzymatic debridement; E, F) secondary dressing; G) 2 months postoperatively; H) 4 month postoperatively

All patients received enzymatic debridement with NexoBrid®.^2^ After the enzymatic debridement, the wound was assessed immediately and therapy decision was made depending on the bleeding pattern (Fig. 1). A red or small punctual bleeding wound, corresponding to a superficial dermal burn wound, was covered with a polylactic acid membrane (Suprathel®, PolyMedics Innovations GmbH, Denkendorf, Germany), a medium to large punctual bleeding pattern, corresponding to a deep dermal burn wound, was covered with Kerecis® Omega3 Wound, and large punctual bleeding pattern with partially exposed subcutaneous tissue, corresponding to a deep dermal to full-thickness burn wound, was treated with split skin grafting (SSG, 0.3 mm, mesh 1:1.5) (Fig. 2).

### Objective and Subjective Wound-Quality Assessment

The wounds of the burn patients were measured during the initial findings according to Wallace rule of nine. The dimensions of covered wound with decellularized fish skin were taken from the operative reports according to the used sizes. Further treatment of Kerecis® covered wounds consisted of a layer of silicone sheet (Mepithel®, Mölnlycke Health Care GmbH, Düsseldorf, Germany). Until the first change of the wound dressing, we used continuous sodium chloride supply trough a cannula fixed with the dressing and a perfusor system. First change of the wound dressing was performed on the third day after the operation and continued every two to three days until epithelization began. Subsequently, wound treatment consisted of daily changed fatty gauze (Jelonet®, Smith & Nephew GmbH, Hamburg, Germany) until complete epithelization was achieved, followed by moisturizing with dexpanthenol until full epithelization.

Time until full epithelization was recorded retrospectively. Time until complete wound healing was defined from the day of operation of the secondary wound dressing (Suprathel®, Kerecis®, or SSG) until the day of full epithelization. For the determination of the scar quality, we used the Vancouver Scar Scale (VSS) and the Patient and Observer Scar Assessment Scale (POSAS). The VSS assesses four scar characteristics including vascularity, height, pliability, and pigmentation. Each characteristic is given a score, which are added together to give an overall score between 0 and 13. The VSS is an observer only score system.^13^ In comparison, the POSAS assesses six characteristics for the patient and observer. The patient scale contains itchiness, painfulness, color, stiffness, thickness, and irregularity compared to the normal skin. The observer scale assesses vascularity, pigmentation, thickness, relief, pliability, and surface area compared to the normal skin.^14^

### Statistical Analysis

The results of the study are presented as the means standard errors of the mean and p-values were calculated using the Mann-Whitney U test when comparing two groups. Statistical significance was considered at a p-value <0.05. Statistical analysis was performed using the IBM® SPSS® software (IBM Deutschland GmbH, Ehningen, Germany).

## Theory

We expected the possibility of scar-free or scar reduced epithelization even in deep dermal burns through the combined use of enzymatic debridement and subsequent wound treatment with decellularized fish skin. Consequently, we postulate a good medium and long-term scar result with high patient satisfaction

## Results

### Patient Data

**Fig. 3:**
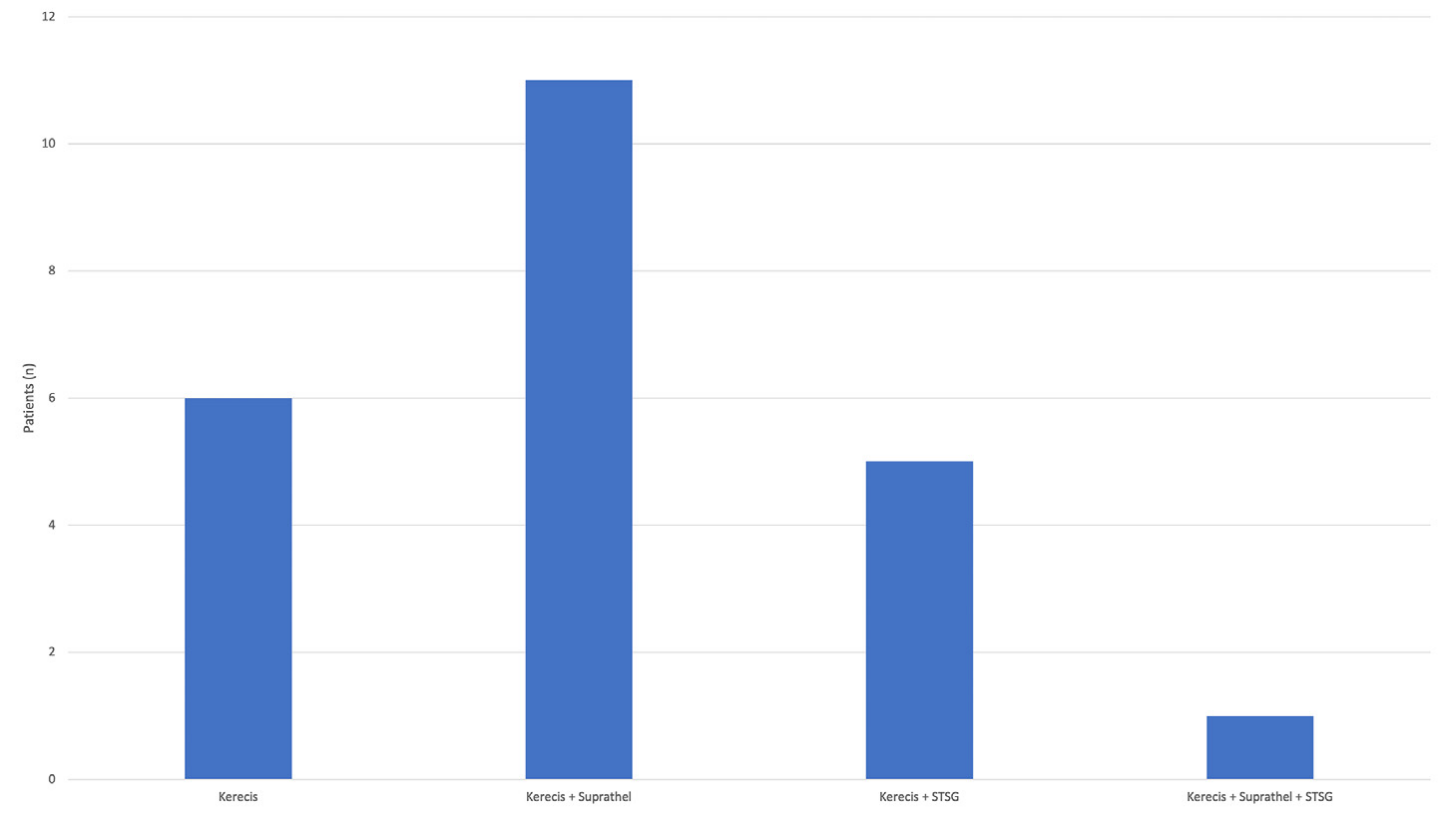
In total, 23 patients were treated. Six patients were treated with decellularized fish skin alone, 11 patients were treated using a combinational therapy of fish skin and polylactide acid membrane (Suprathel®), 1 patient was treated using a combination of decellularized fish skin and split skin transplantation, and 5 patients were treated using combinational therapy involving fish skin, polylactide acid membrane (Suprathel®), and split skin graft. STSG: Split-Thickness Skin Graft.

In total 23 patients, 17 men and 6 women, with a mean age of 34.8 years (2–81 years) were treated. Five patients were lost to follow-up and 18 patients were enrolled in the study with a mean follow-up of 447.6 days. The mean total body surface area (TBSA) was 12.3%. Comorbidities were nicotine abuse (n = 5), drug abuse (n = 2), diabetes mellitus (n = 1), hypertension (n = 2), obstructive lung disease (n = 1), bronchial asthma (n = 1), adipositas (n = 1), hypacusis (n = 1), Raynaud's Syndrome (n = 1), atrial fibrillation (n = 1), paresis of the brachial plexus (n = 1), esophageal reflux (n = 1), hypothyreosis (n = 2), and psychiatric disorders (n = 5). No other comorbidities were known. A total of 7 patients had one or two comorbidities and 3 patients had more than three comorbidities. During hospitalization, 8 patients developed complications with 2 of them devolving multiple complications. Four patients had inhalation trauma, and 2 patients developed septic shock with 1 having rhabdomyolysis, the other having respiratory failure with acute respiratory distress syndrome and acute kidney disease. One patient developed pneumonia and another had *Staphylococcus aureus* bacteremia. All patients received enzymatic debridement and an average area of 247.2 cm^2^ decellularized fish skin was transplanted. Six patients were treated with decellularized fish skin alone, 11 patients using a combination therapy of fish skin and polylactide acid membrane (Suprathel®), 1 patient using a combination of decellularized fish skin and split skin transplantation, and 5 patients using a combinational therapy of fish skin, polylactide acid membrane (Suprathel®), and split skin graft (Fig. 3). Secondary treatment was performed at least 24 h after the enzymatic debridement with a prior soaking phase in polyhexanide.

### Healing Time

**Fig. 4:**
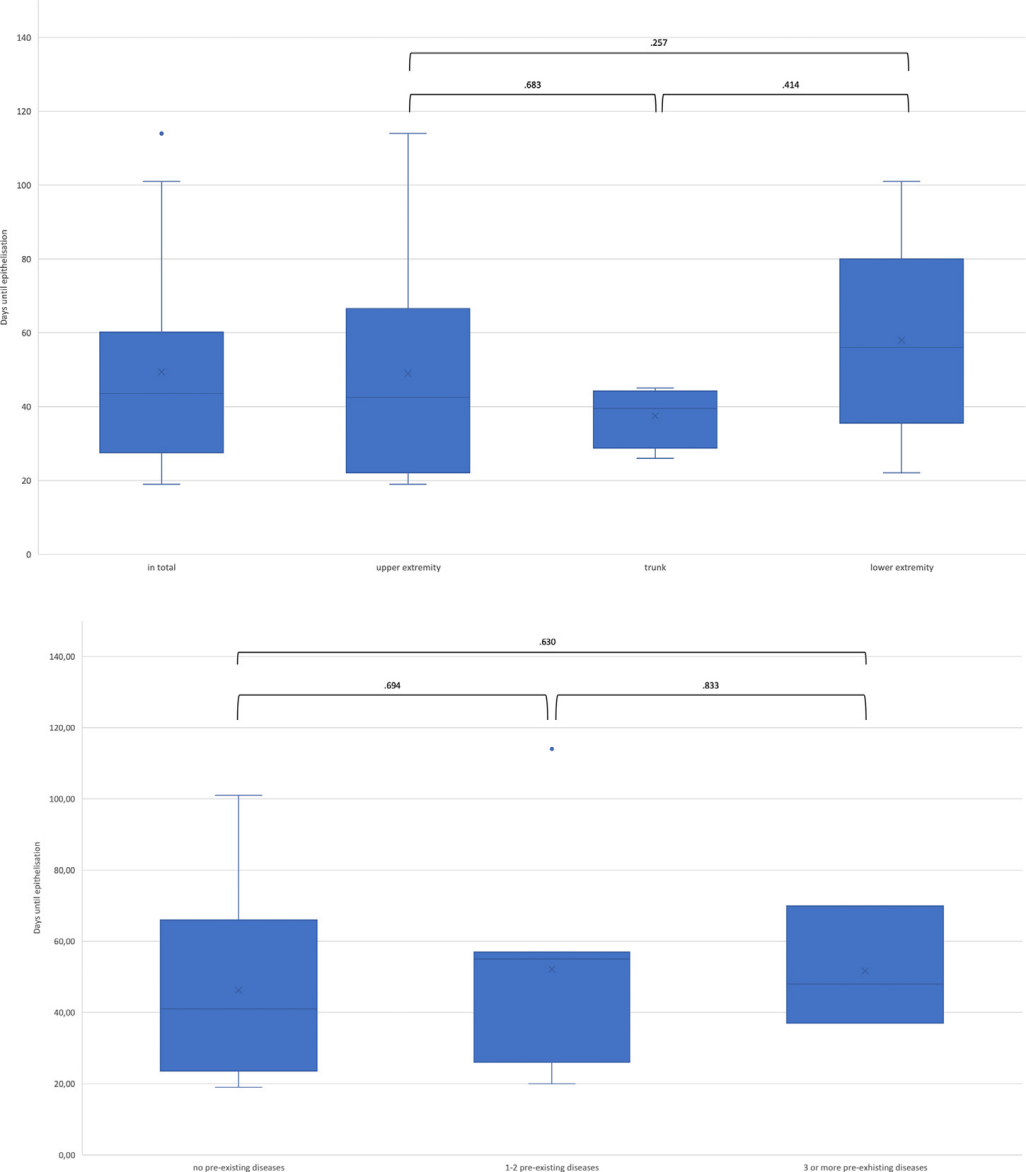

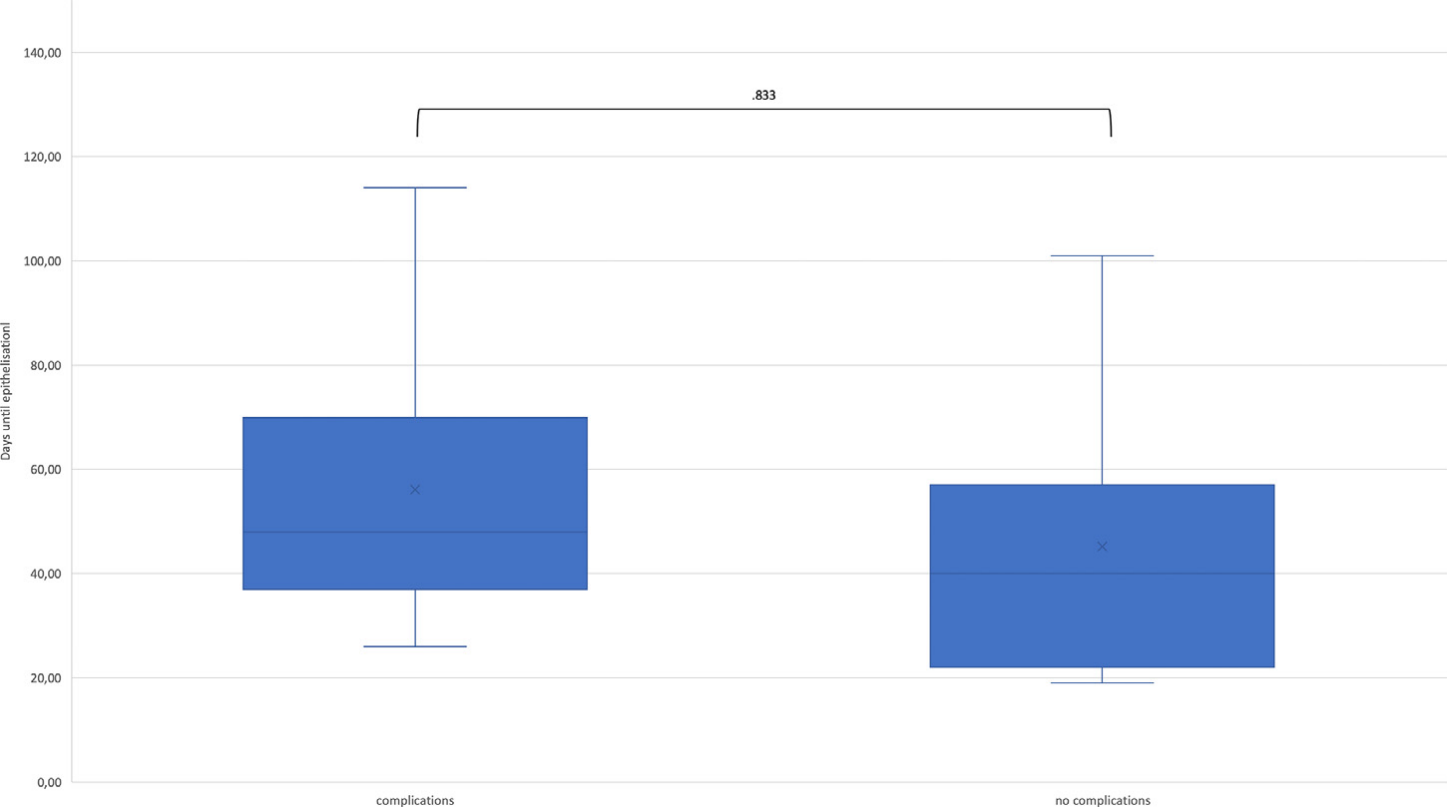
A) Days until epithelization in total and analyses for the upper and lower extremity and the trunk. There was no significant difference between the locations of application. B) Days until epithelization in patient with and without pre-existing diseases. No significant differences were found. C) Days until epithelization in patients suffering from complications during treatment compared to patients without complications. No significant differences were found.

The time taken for the wound to heal completely was 49.4 ± 25.79 days on an average (Fig. 4A). One patient required a secondary split skin graft in the area where decellularized fish skin transplantation was performed. The patient developed septic shock with respiratory failure, acute respiratory distress syndrome, and acute renal failure during the surgical and intensive care treatments. One patient needed secondary amputation of two fingertips and a full-thickness skin graft, which were debrided in the initial treatment with NexoBrid® but not treated with decellularized fish skin. The mean time for the wound healing in the upper extremity was 42 days, 50 days in the lower extremity, and 41 days in the trunk. The average time for complete epithelization in healed patients with scar formation was 63 days on an average.

The mean time for wound healing in patients without pre-existing disease was 46 days, 52 days for patients with one or two pre-existing diseases, and 52 days for patients with three or more pre-existing diseases (Fig. 4B). Patients who developed complications during hospitalization showed a mean time for complete epithelization of 56 days and patients without complications achieved complete epithelization in 45 days (Fig. 4C).

### Scar Quality in Areas Treated with Decellularized Fish Skin

**Fig. 5:**
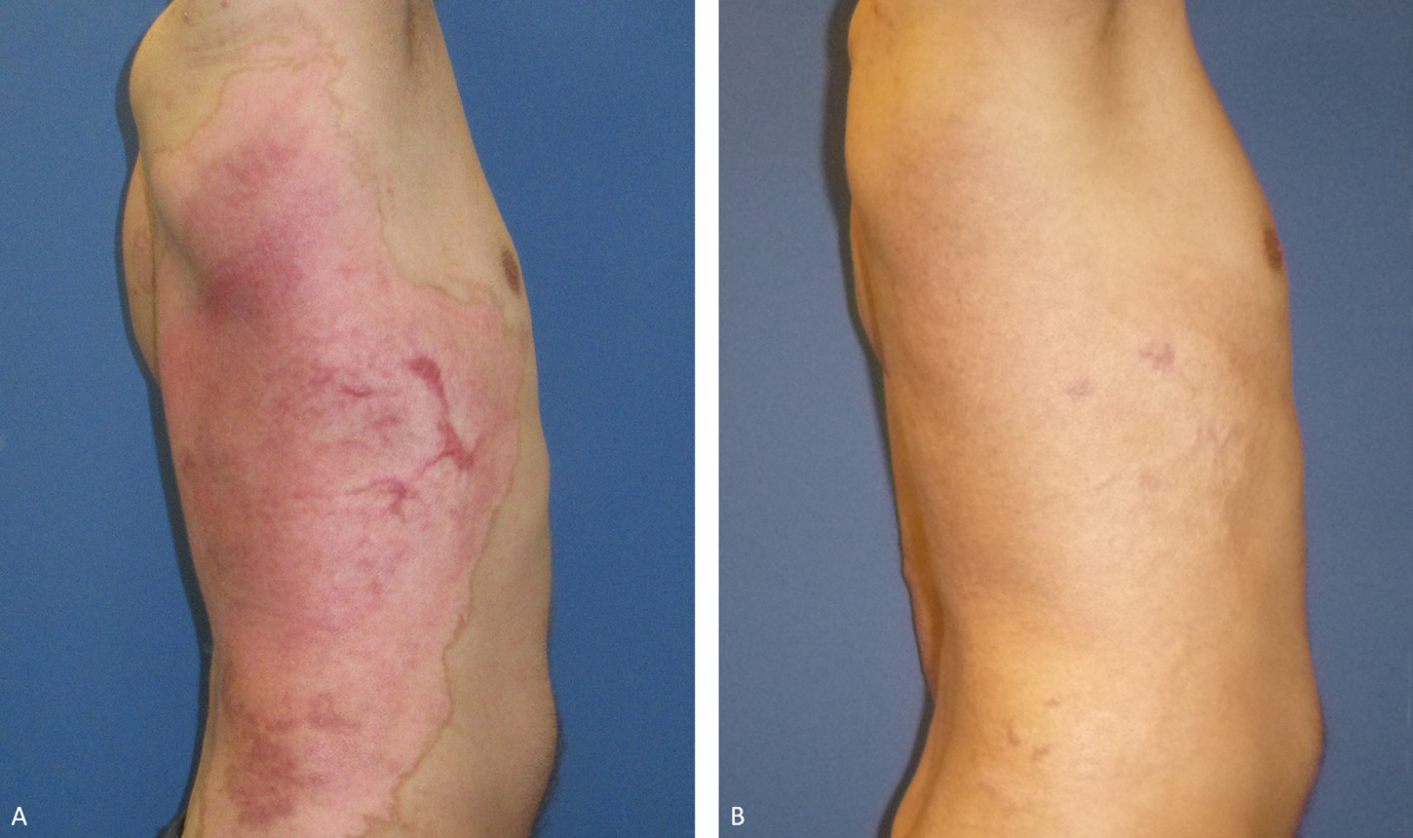
Example of a patient developing minor scarring after hypergranulation during healing time until full epithelization. A) Left: picture 4 month after treatment; B) right: picture 2 years after treatment.

**Fig. 6:**
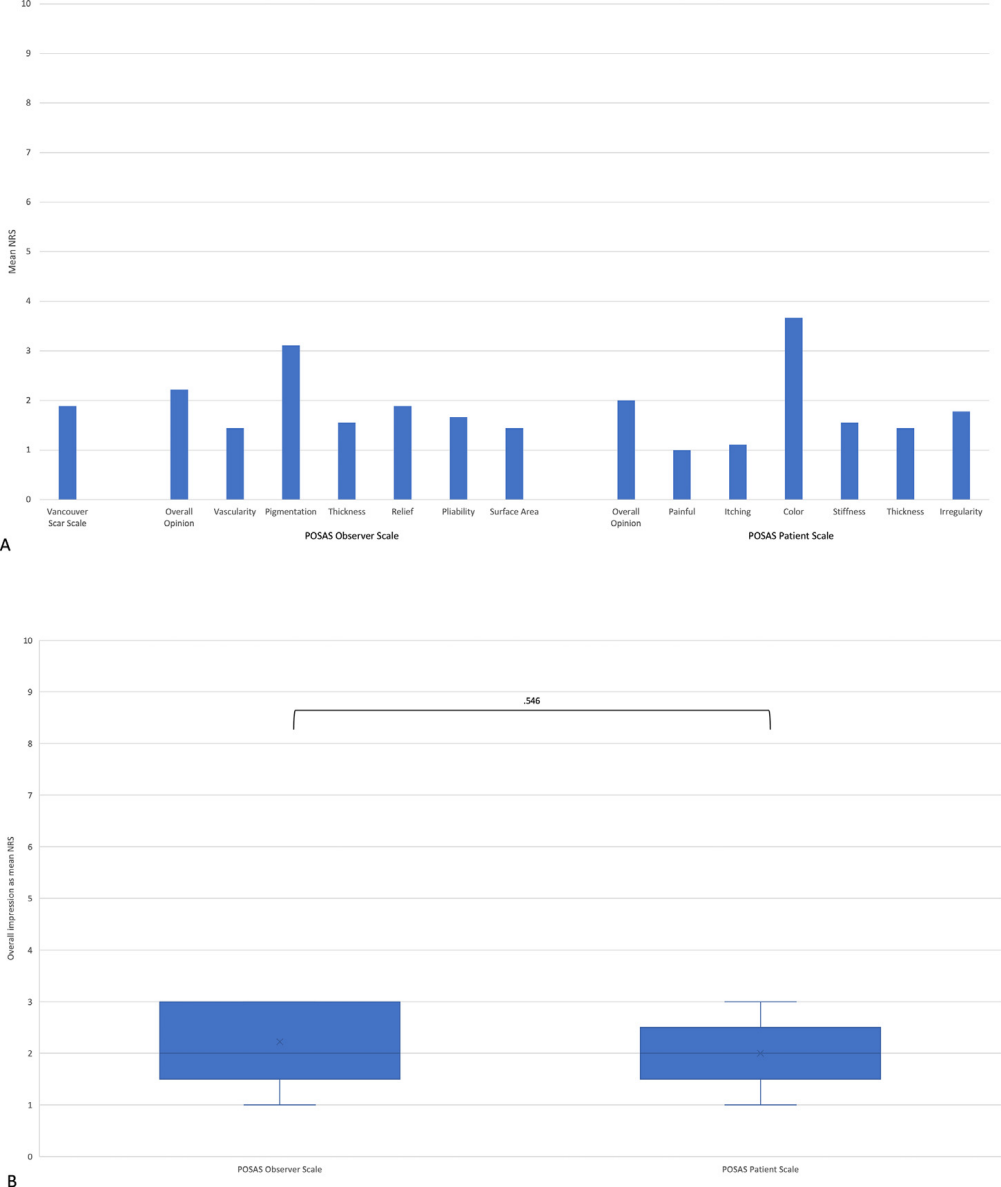
A) Vancouver Scar Scale and POSAS Scale shown as numeric rating scale. The vascularization, pigmentation, thickness, relief, pliability, and the surface area of the scar were observed. The patients rated the pain, itchiness, color, stiffness, thickness, and irregularity of the scar. B) The POSAS observer scale showed an overall impression average of 2.2 ± 0.83. The POSAS patient scale showed an overall impression average 2 ± 0.7. The Vancouver Scar Scale showed an average score of 1.89 ± 1.45. Comparison between the POSAS observer and POSAS patient scales showed no significant differences in the overall opinion. POSAS: Patient and Observer Scar Assessment Scale. NRS: Numeral Rating Scale.

Hypergranulation was observed in 6 patients and minor scarring in 5, none of which led to functional limitations (Fig. 5). Two patients with hypergranulation developed scarring and 4 patients with hypergranulation following scarring had the initial injury at the trunk or lower extremity. No patient showed the development of keloids. Two patients showed temporary vulnerable skin and 2 patients developed pruritus. All patients showed pigment changes, but the skin remained elastic. The POSAS observer scale showed an overall impression average of 2.2 ± 0.83. All patients showed pale-pink vascularization, supple complexion, normal to less relief, and normal to thin skin thickness. Two patients showed hyperpigmentation and 1 exhibited mixed pigmentation; the other patients developed hypopigmentation compared to the uninjured skin. The POSAS patient scale showed an overall impression average of 2 ± 0.7. An average VSS of 1.89 ± 1.45 was obtained (Fig. 6A). Comparison between the POSAS observer and POSAS patient scales showed no significant differences in the overall opinion during scar assessment (Fig. 6A/B 8).

### Development of a Patient-Individualized Modular Treatment

**Fig. 7:**
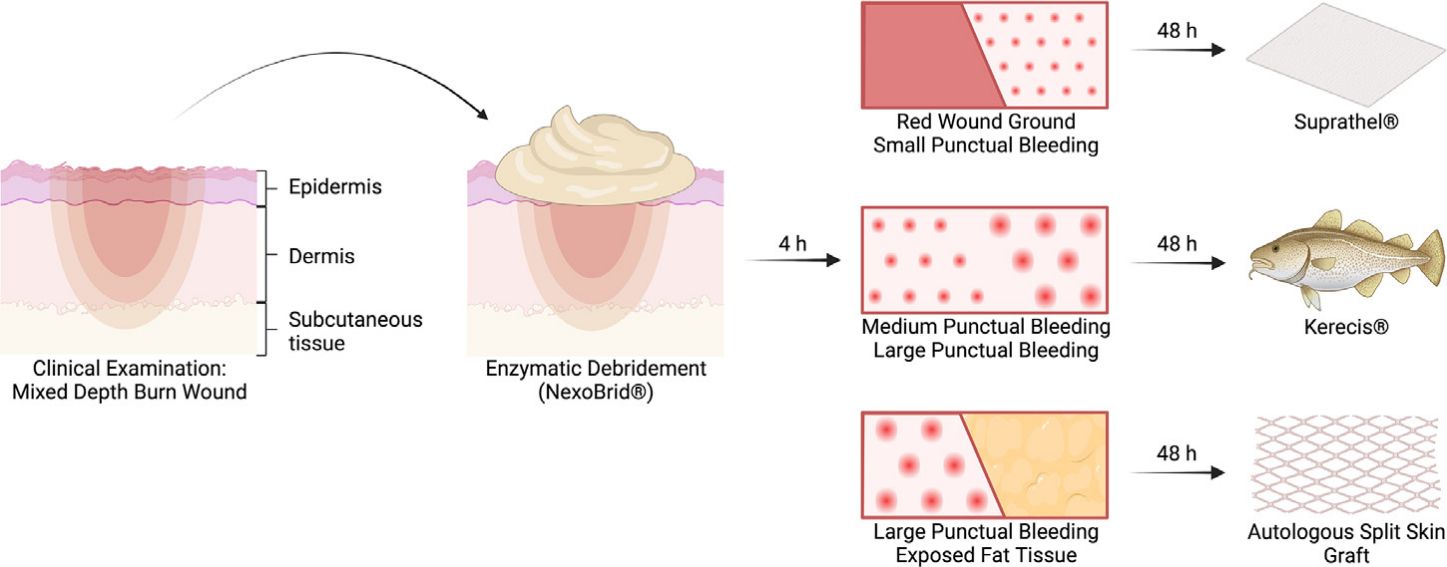
Current therapy algorithm. After the clinical examination, enzymatic debridement is performed for 4 h. Immediately after the removal of the enzyme, the wound assessment is performed and a therapy plan is drawn up based on the bleeding pattern. In the case of superficial dermal injuries with a red wound base and small bleeding points, Suprathel® is applied. If the bleeding pattern is medium to large, the wound is treated by transplantation of decellularized fish skin (Kerecis®). In the case of deeper dermal burn affected areas with visible step formation and exposed fat tissue, split-thickness skin is transplanted.

**Fig. 8:**
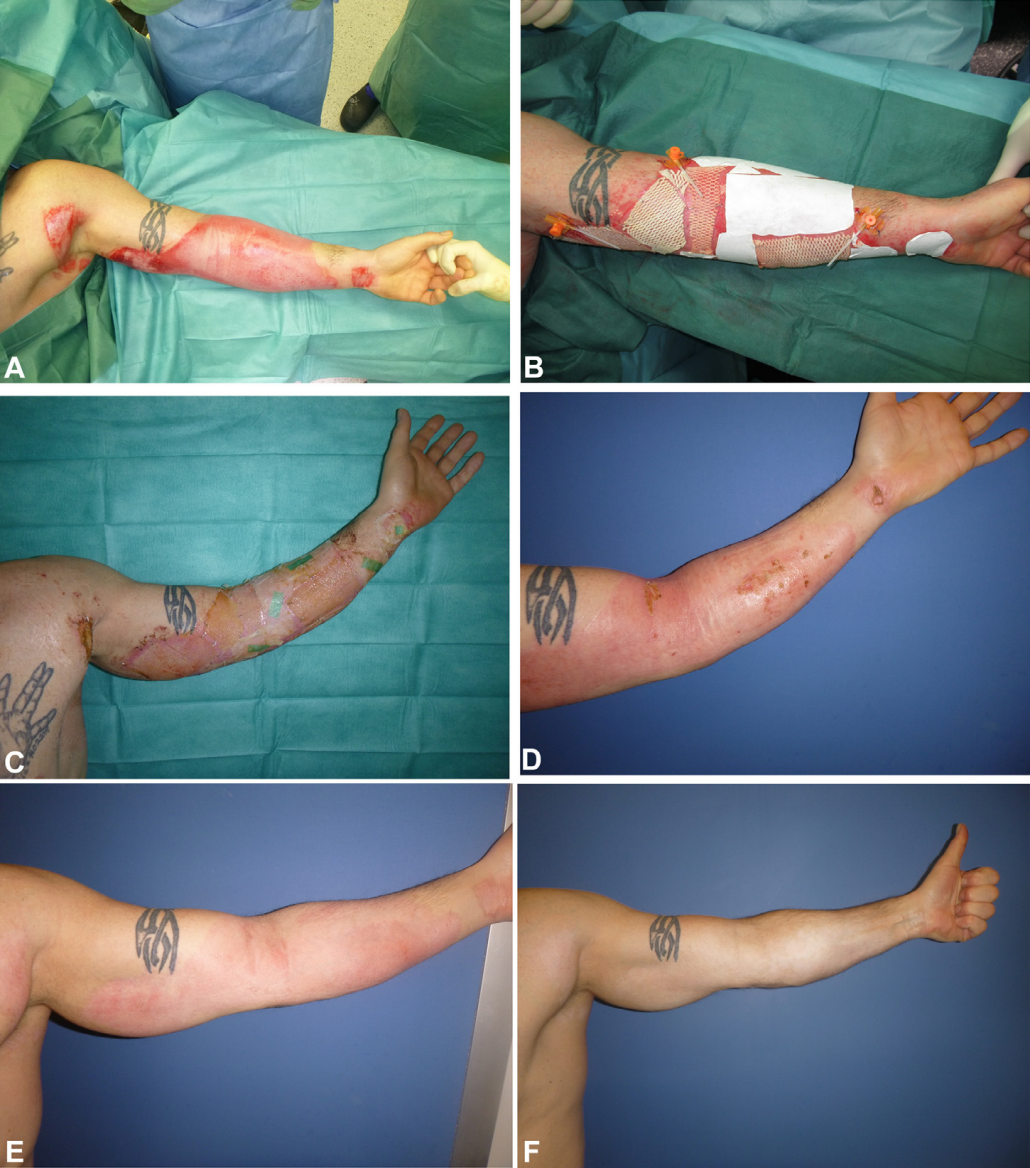
Mixed depth burn wound on the left upper extremity. A) Findings after enzymatic debridement. B) Intraoperative treatment after wound assessment of the bleeding pattern with Kerecis® and Suprathel®. C) Postoperative findings after 14 days. D) Postoperative findings after 25 days. The wound is healed. E) Postoperative findings after 3 months. No scar formation occurred. F) Postoperative findings after 1.5 years. The skin completely recolored with no scar tissue.

Over the last 4 years, our clinic has gone through institutional and individual learning curve for using enzymatic debridement in patients with mixed burns, which we have further developed into a patient-individualized modular treatment principle. After the initial wound assessment and removal of the enzyme, a therapy plan is drawn up using various skin replacement materials or split skin depending on the bleeding pattern. In the case of superficial dermal injuries with a red wound base and small bleeding points, Suprathel® is applied. If the bleeding pattern was medium to large, the wound is treated by transplantation of decellularized fish skin. In case of deeper dermal affected areas with visible step formation, split-thickness skin is transplanted (Fig. 7). After enzymatic debridement, under the postulate of selective debridement, Suprathel® and decellularized fish skin are also used in deeply affected dermis, which leads to a reduction in the total area to be transplanted with skin and thus a reduction in the scar area, while accepting prolonged wound healing. Complete scar-free healing can be achieved in most cases with superficial dermal injuries or at least a reduction in the overall scar area. The epithelialized burn wounds after wound healing showed good elasticity with only slightly hypertrophic scarring compared to split-thickness skin transplants (Fig. 8).

## Discussion

Even if enzymatic debridement has become the standard in the care of severe and mixed degree burns, there is a need for optimization toward standardization of the surgical treatments performed after enzymatic debridement. Depending on the bleeding pattern, transplantation of decellularized fish skin in mid to deep dermal burns showed promising results in clinical application.^15^, ^16^, ^17^

Our retrospective analysis showed satisfying results regarding the overall outcome of the developed scar tissue. The skin quality showed an elastic and supple, complexion with a normal to less skin relief, and normal to thin skin thickness that is comparable to the results reported in a previous retrospective case-control studies.

A comprehensive study with 12 patients included evaluation of the time for healing (95% epithelialization) after enzymatic debridement and secondary dressing with Suprathel®, Kerecis®, or SSG. The average time for wound healing was found to be 22 days after the application of Kerecis®. Nevertheless, our study showed a significantly increased healing time until full epithelization with an average of 50 days compared to 22 days until 95% epithelialization. The comprehensive study used a similar treatment protocol with enzymatic debridement followed by wound assessment based on the bleeding pattern and treatment options included the use of Suprathel®, Kerecis®, or split-thickness skin graft. The mean TBSA of 12.5 ± 9.4% was comparable to our findings (TBSA 12.3 %). The same study showed healing duration of 45.6 ± 6.6 days using Suprathel® and 34.7 ± 12.5 days using split skin transplantation after enzymatic debridement. The evaluation of the wound healing between the different secondary treatments was performed as an intraindividual comparison.^15^ Comparing to our study, the decision was also made based on the observation of the bleeding pattern, which is dependent on the examiner and their experience, and thus therapy decisions postintervention are prone to subjectivity and failure.^18^

No significant differences were found while comparing the healing time in dependence of body region, even if the lower extremity tends to show a longer healing time than the upper extremity or trunk (Fig. 4). Furthermore, in our analysis, we found no statically significant differences in the mean healing time depending on pre-existing diseases or developed complications during the hospitalization (Fig. 4A-C). Assuming that previous illnesses affect the vascular system resulting in prolonged wound healing, the cohort may be too small for a sufficient final assessment of those comorbidities. Moreover, the healing time compared to SSG, with an estimated time of 2-3 weeks after grafting with a range up to 75 days, increased.^19^

Compared to autografting in mid-deep burn wound after enzymatic debridement, our method avoids donor side morbidity, usually healing within 2 to 3 weeks without scars but was described in some cases with a re-epithelization time up to 35 days and risk of an additional hypertrophy scar in up to 28% of the cases.^12^ A combination of granulation and epithelization can lead to spontaneous healing in deep dermal burns after 3 or more weeks, resulting in a scar of poor quality, hypertrophy, and contracture.^20^ This is caused by chronic inflammation among other things.^21^ This inflammatory phase starts several days after the injury and should transition into a proliferative phase, which persists for approximately 6 weeks.^22^ The increasing risk of a scar contraction occasionally is related to the TGF-β pathway.^23^ If the deep dermal end of a wound is involved, healing proceeds with visible scar formation.^24^ Anti-inflammatory characteristics of decellularized fish skin may result in less scar formation than expected when the wound healing time is prolonged.^9^^,^^10^ To obtain sufficient evidence, further studies are required. Even though overall infection rates in autografts and donor site are low, they can cause a loss of the take rate resulting in secondary skin grafting or increased healing time.^12^

Beside the time of healing, the results reported herein are comparable to those of a previous study with satisfactory outcome in the scar quality measured using the POSAS-Score and high patient satisfaction.^15^ However, there are limitations regarding the decision-making for which a substitute should be used according to the bleeding pattern after enzymatic debridement. Previous retrospective photography-based analyses showed that in 50% of the cases evaluated by experts, wounds that were considered for grafting healed spontaneously and 23.5% of the wounds that were expected to heal spontaneously needed a secondary grafting.^25^ Therefore, a laser doppler imaging device can improve the decision-making, reaching a practical and expert consensus for up to 84% of the treatment decision-making.^26^

Although SSG is the gold standard treatment for deep burn wounds, the indication for polylactic membranes and similar dressings (e.g., Suprathel®) differs, based on the depth of the wound and is mainly the standard for superficial burns. However, Suprathel® has also been advocated for mid-deep burn wounds after enzymatic debridement.^2^ In our analysis, decellularized fish skin was used on wounds identified as too deep for treatment with Suprathel®. Therefore, fish skin grafts were expected to be superior on deep dermal burns. A limitation is the clinical assessment of the bleeding pattern after enzymatic debridement as a measurement for wound depth. Different levels of clinical experience and possibly other biases of the deciding clinician are decisive for a further treatment. Hence, final assessment of the proposed treatment regimen continues to be difficult.^18^

Scar assessment showed overall satisfactory results in terms of vascularity, thickness, relief, pliability, surface area, stiffness, and irregularity. Notably, permanent change in skin pigmentation, from hypopigmentation, mixed pigmentation, to hyperpigmentation was observed. The overall impression of the scar between the observers and patients showed no significant differences and a low mean numeric rating scale resulted in good scar quality and the patient-reported level of disturbing pain or itchiness was low.

Limitation of this analysis is the small number of patients in our case series. As described before, Suprathel® was used on more superficial and SSG on deeper wounds compared to decellularized fish skin, which made intraindividual comparison complicated. Moreover, skin condition, properties, and healing rate varied most notably with age.^27^ Furthermore, severe burn injuries and localized burn wound were treated by the combined use of enzymatic debridement and secondary dressing or transplantation. Randomized controlled trials including a higher number of cases and standardized wound assessment protocol would be necessary.

## Conclusion

In conclusion, a combined treatment involving enzymatic debridement and secondary dressing with decellularized fish skin, polylactide membrane, or split skin grafts allows for a more individualized therapy for mixed depth burn wounds. Fish skin was found to provide satisfactory results in terms of the overall outcome of the developed scar tissue and could lead to a reduction in the area requiring autologous transplantation. Therefore, it also led to a reduction in the wound surface through an absence of donor side morbidity with possible additional complications. However, time until complete epithelization was increased. A potential improvement in the quality of life could possibly be reached using fish skin grafts as indicated by our patient satisfaction scores.

## Disclosure

The authors have no conflicts of interest to disclose. No funding was received for this article.

## Funding sources

This research did not receive any specific grant from funding agencies in the public, commercial, or not-for-profit sectors.

## Credit author statement

GF, AB, BS, and JB treated all patients and designed the study. GF, AB, and BS designed and conducted all clinical tests and examined the patients. All author provided help and advice on GF and AB analyzing the data. GF and AB drafted the work and wrote the manuscript. All authors contributed to editorial changes in the manuscript. All authors read and approved the final manuscript.

## “Ethical Approval” and “Patient consent Statement for photo publication”

Data from patients with mixed dermal burn wounds receiving enzymatic debridement followed by wound coverage with decellularized fish skin were collected retrospectively. Written consent was provided from all patients. The study is approved by the Ethics Committee at the Medical Faculty of the RWTH Aachen University.
